# Supplementary material for: Genetic evidence for a large overlap and potential bidirectional causal effects between resilience and well-being
Source: Neurobiol Stress. 2021 Mar 14;14:100315. doi: 10.1016/j.ynstr.2021.100315 (PMC8010858; doi:10.1016/j.ynstr.2021.100315)
Supplement: Multimedia component 1 [file mmc1.docx]

**Supplementary information**

**Table S1.**

*Number of participants per zygosity and sex for every variable.*

|  | Resilience 1 | |  |  | Well-being 1 | | |  | Resilience 2 | |  |  | Well-being 2 | | |  |
| --- | --- | --- | --- | --- | --- | --- | --- | --- | --- | --- | --- | --- | --- | --- | --- | --- |
|  | T 1 | T 2 | S m | S f | T 1 | T 2 | S m | S f | T 1 | T 2 | S m | S f | T 1 | T 2 | S m | S f |
| MZF | 769 | 717 | 127 | 187 | 795 | 747 | 126 | 198 | 1367 | 1259 | 144 | 220 | 1674 | 1508 | 171 | 267 |
| MZM | 323 | 264 | 74 | 71 | 333 | 274 | 76 | 74 | 506 | 441 | 69 | 99 | 649 | 551 | 87 | 121 |
| DZF | 412 | 336 | 67 | 86 | 433 | 354 | 70 | 92 | 708 | 574 | 82 | 124 | 891 | 709 | 96 | 142 |
| DZM | 178 | 128 | 35 | 46 | 184 | 125 | 35 | 47 | 293 | 224 | 40 | 66 | 394 | 283 | 49 | 78 |
| DZO | 392 | 358 | 82 | 110 | 405 | 377 | 83 | 119 | 629 | 621 | 89 | 147 | 914 | 813 | 105 | 183 |
|  |  |  |  |  |  |  |  |  |  |  |  |  |  |  |  |  |
| Total | 2074 | 1803 | 385 | 500 | 2150 | 1877 | 390 | 530 | 3503 | 3119 | 424 | 656 | 4522 | 3864 | 508 | 791 |

*Note*: T1= twin 1, T2= twin 2, Sm= sibling male, Sf= sibling female.

**Table S2.**

*The stressful life events included in the questionnaire at both time points*.

| Time 1 |  | Time 2 |
| --- | --- | --- |
| 1.   Death of life partner, | | 1.  Financial problems, |
| 2.   Death of father, | | 2.  Job loss, |
| 3.   Death of mother, | | 3.  Drop out of education, |
| 4.  Death of child, | | 4.  Relationship problems with partner, |
| 5.   Death of sibling, | | 5.  Relationship problems with child, |
| 6.   Death of other loved one, | | 6.  Relationship problems with other loved one, |
| 7.  Serious disease of yourself, | | 7.  Getting hospitalized, |
| 8.   Serious disease of life partner, | | 8.  Serious illness yourself, |
| 9.   Serious disease of child, | | 9.  Serious illness partner, |
| 10. Serious disease of other loved one, | | 10. Serious illness child, |
| 11.  End of relation, | | 11. Serious illness parent, |
| 12.  Traffic accident, | | 12. Serious illness other loved one, |
| 13.  Violent crime, | | 13. Death of partner, |
| 14.  Sexual crime, | | 14. Death of child, |
| 15.  Theft, | | 15. Death of other loved one, |
| 16.  Getting fired. | | 16.  Traffic accident, |
|  |  | 17.  Theft, |
|  |  | 18.  Violent crime, |
|  |  | 19.  Sexual crime. |

Table S3.
*Results of the psychometric model constraining the genetic and environmental correlation.*

| Base | Comparison | -2LL | df | AIC | ΔLL | Δ df | p |
| --- | --- | --- | --- | --- | --- | --- | --- |
| **AE** |  | 176449.79 | 27054 | 122341.79 |  |  |  |
| AE | rA=0 | 176731.08 | 27055 | 122621.08 | 281.29 | 1 | 3.93E-63 |
| AE | rE=0 | 176966.24 | 27055 | 122856.24 | 516.46 | 1 | 2.50E-114 |

*Note*: rA= genetic correlation, rE= environmental correlation.

Table S4.

*Results of the longitudinal twin model fitting for resilience at baseline and well-being a few years later.*

| Base | Comparison | ep | -2LL | df | AIC | ΔLL | Δdf | p |
| --- | --- | --- | --- | --- | --- | --- | --- | --- |
| **AE** |  | 16 | 93841.40 | 14431 | 64979.40 |  |  |  |
| AE | rAf=0 | 15 | 93946.52 | 14432 | 65082.52 | 105.13 | 1 | <.0001 |
| AE | rAm=0 | 15 | 93867.60 | 14432 | 65003.60 | 26.20 | 1 | <.0001 |
| AE | rEf=0 | 15 | 93863.40 | 14432 | 64999.37 | 21.97 | 1 | <.0001 |
| AE | rEm=0 | 15 | 93853.11 | 14432 | 64989.12 | 11.73 | 1 | <.0001 |

*Note*: ep= estimated parameters, rAf= genetic correlation females, rAm= genetic correlation males, rEf= environmental correlation females, rEm= environmental correlation males.

Table S5.

*Results of the longitudinal twin model fitting for well-being score at baseline and resilience a few years later.*

| base | comparison | ep | -2LL | df | AIC | ΔLL | Δdf | p |
| --- | --- | --- | --- | --- | --- | --- | --- | --- |
| AE |  | 16 | 86832.88 | 12633 | 61566.88 |  |  |  |
| AE | rAf=0 | 15 | 86909.84 | 12634 | 61641.84 | 76.96 | 1 | <.0001 |
| AE | **rAm=0** | 15 | 86837.44 | 12634 | 61569.44 | 4.55 | 1 | **.0329** |
| AE | rEf=0 | 15 | 86855.64 | 12634 | 61587.64 | 22.76 | 1 | <.0001 |
| AE | rEm=0 | 15 | 86854.52 | 12634 | 61586.52 | 21.63 | 1 | <.0001 |

Note: ep= estimated parameters, rAf= genetic correlation females, rAm= genetic correlation males, rEf= environmental correlation females, rEm= environmental correlation males.

Table S6.

*Results of the MR-DoC model fitting for the different time points.*

| base | comparison | ep | -2LL | df | AIC | ΔLL | Δdf | p |
| --- | --- | --- | --- | --- | --- | --- | --- | --- |
| **Time 1** |  |  |  |  |  |  |  |  |
| WB -> RES |  | 35 | 22796.60 | 8442 | 5912.6 |  |  |  |
| WB -> RES | g1=0 | 34 | 22898.07 | 8443 | 6012.1 | 101.5 | 1 | 7.27E-24 |
| **Time 2** |  |  |  |  |  |  |  |  |
| WB -> RES |  | 35 | 30263.71 | 11243 | 7777.707 |  |  |  |
| WB -> RES | g1=0 | 34 | 30560.39 | 11244 | 8072.391 | 296.684 | 1 | 1.74E-66 |

Note: WB= well-being, RES= resilience, g1= causal effect, ep= estimated parameters, df= degrees of freedom.

Table S7.

*Estimates of the MR-DoC models for the different time points.*

| **Standardized** |  | **Time 1:**  **WB -> Resilience** | | **Time 2:**  **WB -> Resilience** | |
| --- | --- | --- | --- | --- | --- |
|  |  |  |  |  |  |
|  | name | Estimate | Std.Error | Estimate | Std.Error |
| Causal effect | g1 | 0.337 | 0.033 | 0.443 | 0.025 |
| PGS exposure | b1 | 0.077 | 0.022 | 0.098 | 0.017 |
| PGS outcome | b2 | 0.109 | 0.019 | 0.069 | 0.016 |
| A effect exposure | ab | 0.638 | 0.032 | 0.581 | 0.025 |
| E effect exposure | eb | 0.795 | 0.022 | 0.816 | 0.016 |
| A effect outcome | as | 0.530 | 0.029 | 0.502 | 0.022 |
| E effect outcome | es | 0.659 | 0.018 | 0.673 | 0.015 |
| PGS to PGS | x | 1.001 | 0.012 | 1.001 | 0.012 |
| Genetic correlation | ra | 0.521 | 0.090 | 0.159 | 0.082 |

Note: WB= well-being.

Table S8.

*Model fit of models with various fixed rE values.*

| **Time 1** | rE | parameters | -2LL | df | AIC | g1 | R2 (%) |
| --- | --- | --- | --- | --- | --- | --- | --- |
|  | 0 | 35 | 8442 | 22796.6 | 5912.6 | 0.337 | 11.36% |
|  | 0.1 | 35 | 8442 | 22796.6 | 5912.6 | 0.2536 | 6.43% |
|  | 0.2 | 35 | 8442 | 22796.6 | 5912.6 | 0.168 | 2.81% |
|  | 0.3 | 35 | 8442 | 22796.6 | 5912.6 | 0.076 | 0.58% |
|  | 0.4 | 35 | 8442 | 22797.1 | 5913.08 | 0 | 0.00% |
|  | 0.5 | 35 | 8442 | 22811.3 | 5927.3 | 0 | 0.00% |
|  | 0.8 | 35 | 8442 | 23106.7 | 6222.7 | 0 | 0.00% |
| **Time 2** | rE | parameters | df | -2LL | AIC | g1 | R2 (%) |
|  | 0 | 35 | 11243 | 30263.7 | 7777.71 | 0.443 | 19.62% |
|  | 0.1 | 35 | 11243 | 30263.7 | 7777.71 | 0.36 | 12.96% |
|  | 0.2 | 35 | 11243 | 30263.7 | 7777.71 | 0.275 | 7.56% |
|  | 0.3 | 35 | 11243 | 30263.7 | 7777.71 | 0.184 | 3.39% |
|  | 0.4 | 35 | 11243 | 30263.7 | 7777.71 | 0.083 | 0.69% |
|  | 0.5 | 35 | 11243 | 30265.1 | 7779.07 | 1.1E-14 | 0.00% |
|  | 0.8 | 35 | 11243 | 30832.1 | 8346.13 | 3.9E-10 | 0.00% |

**
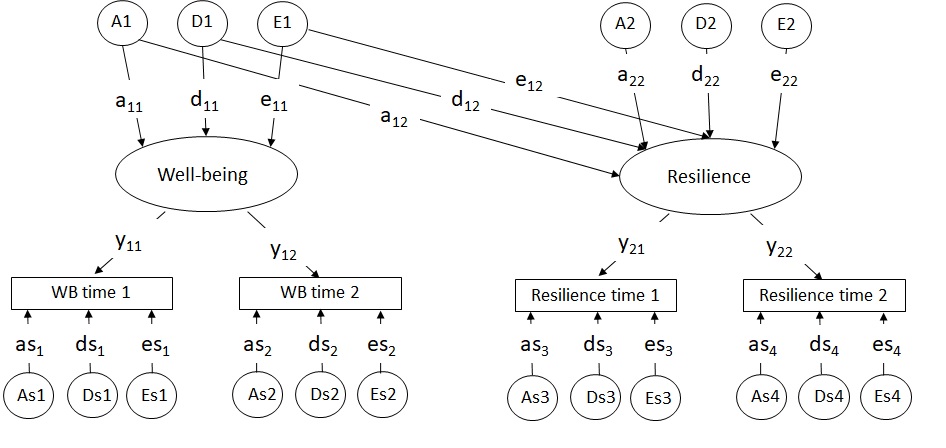
**

**Figure S1.** Longitudinal measurement model. WB= well-being, A= common additive genetic effect, E= common unique environmental effects, As= time-specific additive genetic effect, Es= time-specific environmental effect.

**
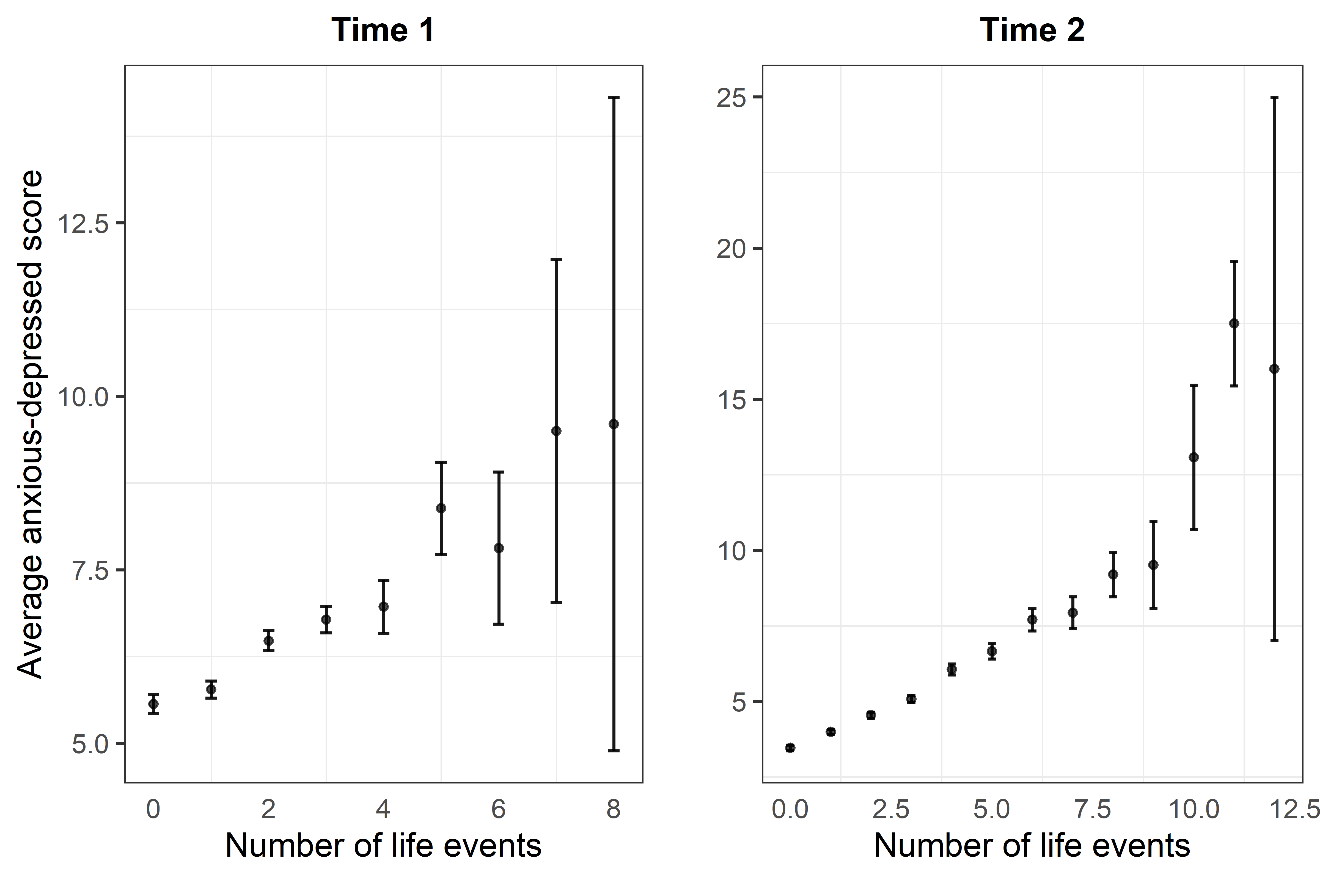
**

**Figure S2.** Association between the number of life events and the anxious depressed score.

**
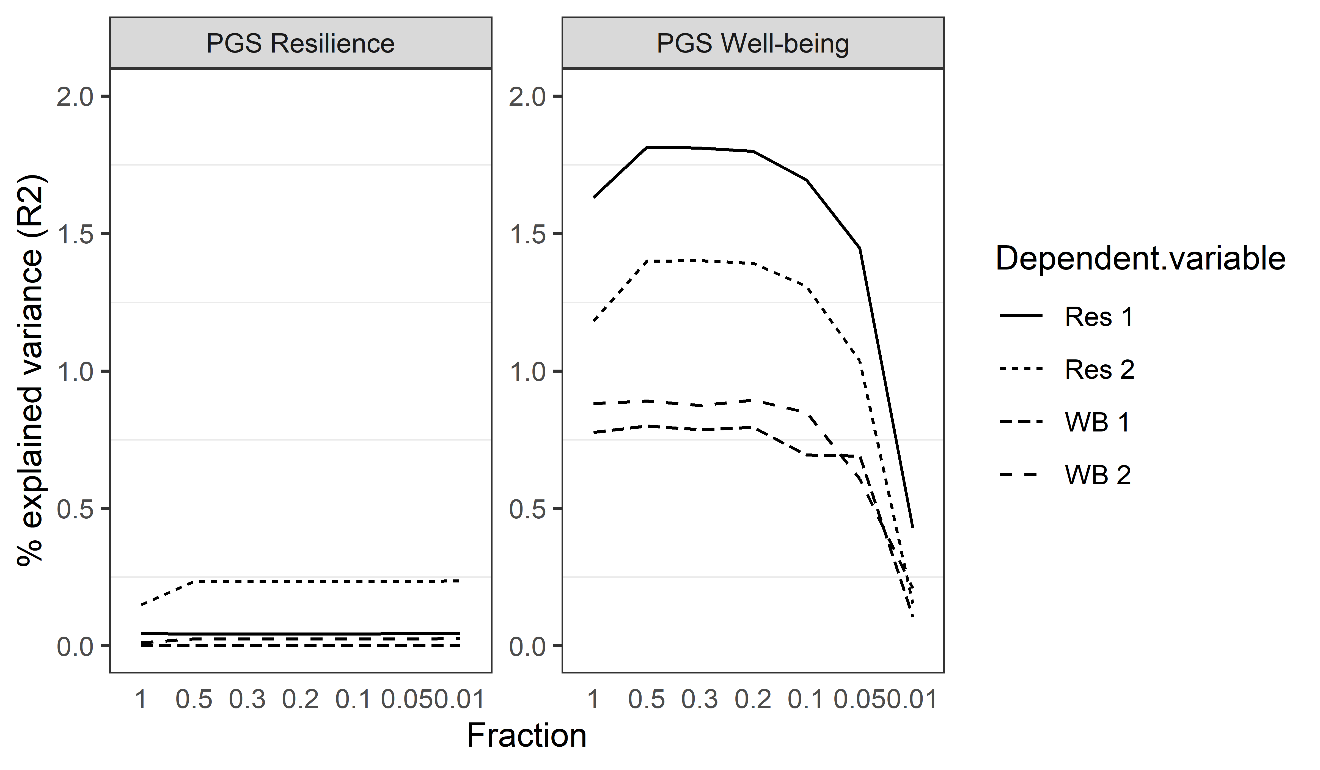
**

**Figure S3**. Explained variance by the resilience and well-being PGS for the different fractions of SNPs included.

**
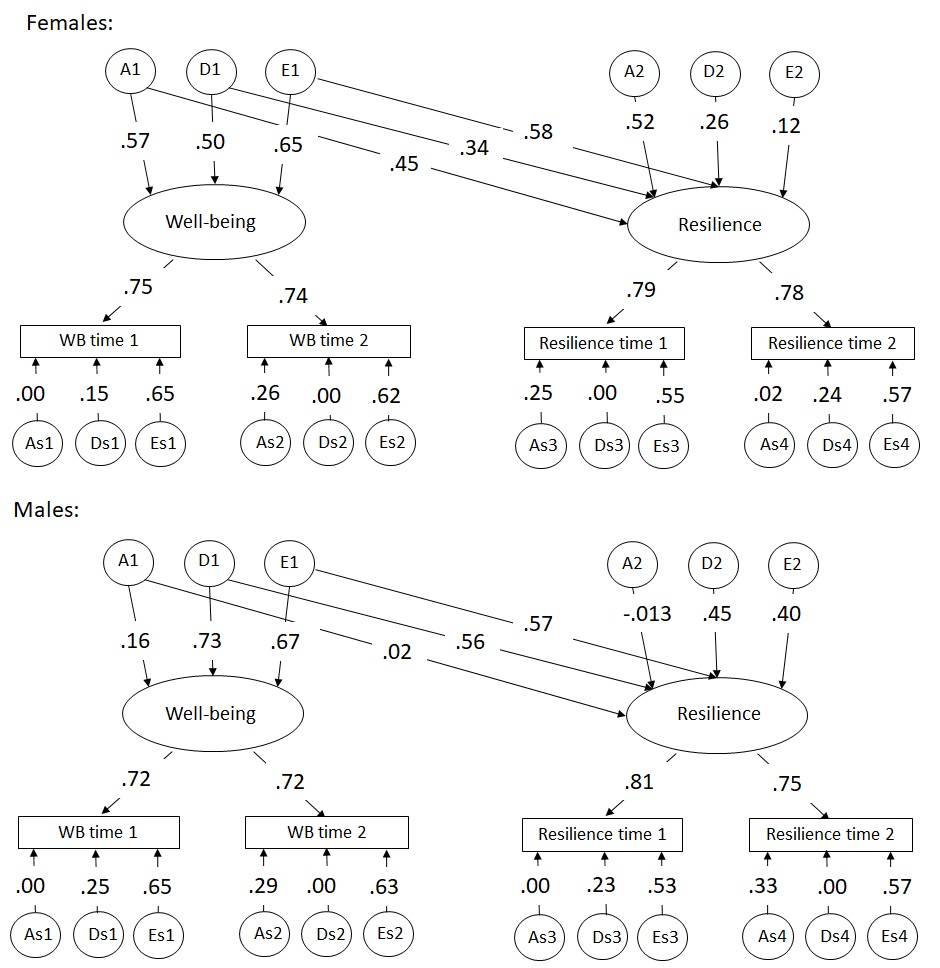
**

**Figure S4.** Path estimates of the full common pathway model of resilience and well-being for females and males. WB= well-being, A= common additive genetic effect, E= common unique environmental effects, As= time-specific additive genetic effect, Es= time-specific environmental effect.
